# Looking beyond GWAS: allele-specific transcription factor binding drives the association of *GALNT2* to HDL-C plasma levels

**DOI:** 10.1186/s12944-016-0183-x

**Published:** 2016-01-27

**Authors:** Marco Cavalli, Gang Pan, Helena Nord, Claes Wadelius

**Affiliations:** Science for Life Laboratory, Department of Immunology, Genetics and Pathology, Uppsala University, Uppsala, Sweden

**Keywords:** AS-SNPs, GWAS, HDL-C, GALNT2

## Abstract

**Background:**

Plasma levels of high-density lipoprotein cholesterol (HDL-C) have been associated to cardiovascular disease. The high heritability of HDL-C plasma levels has been an incentive for several genome wide association studies (GWASs) which identified, among others, variants in the first intron of the *GALNT2* gene strongly associated to HDL-C levels. However, the lead GWAS SNP associated to HDL-C levels in this genomic region, rs4846914, is located outside of transcription factor (TF) binding sites defined by chromatin immunoprecipitation followed by DNA sequencing (ChIP-seq) experiments in the ENCODE project and is therefore unlikely to be functional. In this study we apply a bioinformatics approach which rely on the premise that ChIP-seq reads can identify allele specific binding of a TF at cell specific regulatory elements harboring allele specific SNPs (AS-SNPs). EMSA and luciferase assays were used to validate the allele specific binding and to test the enhancer activity of the regulatory element harboring the AS-SNP rs4846913 as well as the neighboring rs2144300 which are in high LD with rs4846914.

**Findings:**

Using luciferase assays we found that rs4846913 and the neighboring rs2144300 displayed allele specific enhancer activity. We propose that an inhibitor binds preferentially to the rs4846913-C allele with an inhibitory boost from the synergistic binding of other TFs at the neighboring SNP rs2144300. These events influence the transcription level of *GALNT2*.

**Conclusions:**

The results suggest that rs4846913 and rs2144300 drive the association to HDL-C plasma levels through an inhibitory regulation of *GALNT2* rather than the reported lead GWAS SNP rs4846914.

## Introduction

Low plasma levels of high-density lipoprotein cholesterol (HDL-C) are strongly associated with high risk of coronary artery disease (CAD). Low HDL-C levels together with high levels of low-density lipoprotein cholesterol (LDL-C) and triglycerides (TG) are common features of dyslipidemia and combined hyperlipidemia [1]. Plasma levels of HDL-C have a heritability of 40–60 % [2] and several genome wide association studies (GWAS) [3, 4] have been performed to pinpoint the associated genetic variants. Variations in the first intron of the *GALNT2* gene on chromosome 1q42 have been associated with HDL-C and TG plasma levels in several studies [3, 5]. *GALNT2* encodes for N-actetylgalactosaminyltransferase 2, an enzyme involved in the O-linked glycosylation of proteins [6]. It glycosylates apolipoprotein C-III which is an inhibitor of lipoprotein lipase (LPL). LPL hydrolyses plasma triglycerides in very low-density lipoprotein (VLDL) and chylomicrons [7] that influence HDL-C and triglyceride levels. Overexpression of *Galnt2* in mouse models lowered HDL-C while knock-down of *Galnt2* resulted in higher HDL-C levels [4] and *GALNT2* expression in liver is correlated to the lead GWAS SNP (rs4846914) associated to HDL-C plasma levels [8].

The SNP reported with the strongest association in GWAS rarely is the functional one. Previous studies have shown that SNPs associated to disease are enriched in regulatory elements [9] and it is generally assumed that the ones driving the association affect the activity of a gene and that this is mediated by a transcription factor (TF) binding with different strength to the two alleles, variants, of the SNPs. We have therefore scanned public data to find such SNPs and we utilized the TF binding data from ENCODE ChIP-seq experiments [10] in the epithelial hepatocellular carcinoma cell line HepG2 to identify cis-acting regulatory variants that are relevant for liver function (see Methods). SNPs in transcription factor binding sites (TFBS) that showed an allele specific binding (AS-SNPs) are suggested to be the drivers of the associations observed in GWAS studies. The lead SNP associated to HDL-C plasma levels was not located in a regulatory element and therefore not likely to mediate the association so in this study we tested the functional activity of an AS-SNP in high linkage disequilibrium (LD) with the lead GWAS SNP.

## Methods

### AS-SNPs definition

AS-SNPs were defined by our in house AS-SNPs selection pipeline which identifies heterozygous SNPs that show a statistically significant difference in ChIP-seq read count on the two alleles. The pipeline follows these steps: (**1**) publicly available raw reads (.fastq) from ChIP-seq for several TFs in HepG2 were obtained from the ENCODE project database (ftp://hgdownload.cse.ucsc.edu/goldenPath/hg19/encodeDCC/) and were aligned to the reference human genome (G1) UCSC hg19 assembly based on the Genome Reference Consortium Human genome build 37 (GRCh37) but excluding random and unplaced contigs and to an HepG2-specific alternative genome (G2), built using the FastaAlternateReferenceMaker GATK utility that generates an alternative reference sequence replacing the reference bases at variation sites with the bases supplied by a SNPs collection. SNP calls were made from pooled short-read datasets in house ChIP-seq data plus ENCODE genomic HepG2 reads and were validated using a genome-wide genotyping array. (**2**) Reads mapped specifically to G1 or G2 were counted at the heterozygous SNPs. SNPs with no reads mapped on G1 or G2 were discarded. (**3**) To determine whether the G1/G2 read counts difference was statistically significant a binomial test was applied against the null hypothesis of an equal G1:G2 coverage. After correcting for multiple testing (Benjamini & Hochberg or FDR), AS-SNPs with *P* < 0.05 were selected. (**4**) AS-SNPs were then intersected with the 1,000 Genomes SNPs collection (1000 Genomes project, phase1_release_v3.20101123) in order to retrieve AFs. (**5**) Extensive filtering of the selected AS-SNPs was performed to remove AS-SNPs that were in centromeric or telomeric regions (UCSC Gap table ± 1 Mb), blacklisted ENCODE regions (±100 bp) or CNVs. (**6**) Pruned AS-SNPs selections were finally intersected with collections of GWAS associated to liver-related and metabolic relevant traits and SNPs in LD (r^2^ > 0.8) with GWAS (proxy SNPs calculated via SNAP tool [11]) to select candidate functional AS-SNPs for experimental validation.

### Cell cultures

HepG2 cells were cultured in RPMI 1640 medium supplemented with 10 % non-inactivated FBS, L-glutamine and a solution stabilized, with 10,000 units penicillin and 10 mg streptomycin/mL, sterile-filtered, BioReagent, suitable for cell culture (Sigma-Aldrich) at 37 °C with 5 % CO_2_.

### Construction of cloning plasmids and luciferase reporter assays

All the luciferase expression constructs were built based on pGL4.23 from Promega. The ccdB expression cassette was inserted into KpnI and EcoRV sites of pGL4.23 to construct pGL4.23-ccdB, which was used as a basal vector to diminish false positive signal during the cloning process. Genomic sequences surrounding AS-SNPs were amplified by Phusion Hot Start Flex DNA polymerase (NEB) using HepG2 genomic DNA as template. The amplified fragments were purified by the QIAquick Gel Extraction Kit (QIAGEN) and inserted upstream of the minimal promoter sequence of pGL4.23 by SLiCE cloning methods [12]. To test both of the alleles of the AS-SNP, multiple individual clones were picked and subjected to Sanger sequencing. HepG2 cells were transfected one day after plating with approximately 90 % confluence in 96-well plate. All the transfection reactions were carried out with the X-tremeGENE HP DNA transfection reagent (Roche). To normalize the transfection and lysis efficiency each well was transfected with 100 ng of firefly luciferase reporter vector harboring respective AS-SNP alleles together with 1 ng of renilla luciferase reporter vector pGL4.74,. Twenty-four hours after transfection, the cells were harvested and lysed in 1X passive lysis buffer (Promega) on a rocking platform for 45 min at room temperature. Firefly and luciferase activity were measured by Dual-Luciferase® Reporter (DLR™) Assay System (Promega) on an Infinite® M200 pro reader (TECAN) following instructions provided by the manufacturer. The ratios of firefly luciferase activity to renilla luciferase activity were calculated and expressed as Relative Luciferase Units (RLU) in the figures. All data came from six replicate wells, and *p*-values comparing RLU difference between AS-SNPs alleles were calculated using two-tailed *t*-test.

### EMSA

Nuclear extracts were prepared from HepG2 cells using the NucBuster™ Protein Extraction kit (Novagen) and the amount was verified via the Qubit® Protein Assay Kit (Life Technologies). Oligonucleotide probes were designed with both alleles of rs4846913 flanked by 14 bp in both a cold and 5'-biotinylated form (IDT). Biotinylated and un-biotinylated oligonucleotides were annealed with the reverse complementary oligonucleotide (95 °C to 25 °C temperature stepdown). For the binding reaction, 3–6 μg of the nuclear extract were incubated with 200 fmol of each biotinylated dsDNA probe for 40 min on ice in 10 mM TrisHCl, 30 mM KCl, 1 mM DTT, 1 μg of Poly (dI-dC), 7.5 % glycerol, 0.063 % NP-40, 2 mM MgCl2, and 0.1 mM EDTA. Samples were electrophoresed in Criterion™ 5 % TBE Precast Gels (Bio-rad), and electro-transferred into a Genescreen plus™ hybridization transfer membrane (Perkin Elmer). DNA-protein complexes were cross-linked using UV-light and detected by chemiluminescence using LightShift® Chemiluminescent EMSA Kit (Thermo Scientific).

## Results and Discussion

We identified a collection of AS-SNPs showing differential allele binding of TFs based on ChIP-seq experiments performed in HepG2 cells. The preferential TF binding to a specific allele at heterozygous position in the genome was used to pinpoint putative functional regulatory elements. To avoid a bias toward the reference human genome the ChIP-seq reads were aligned to the maternal and paternal genomes in HepG2. The difference in the number of reads mapping to the two alleles was calculated and tested for statistical significance in order to identify suitable candidate regulatory elements for experimental validation (see Methods).

**Fig. 1:**
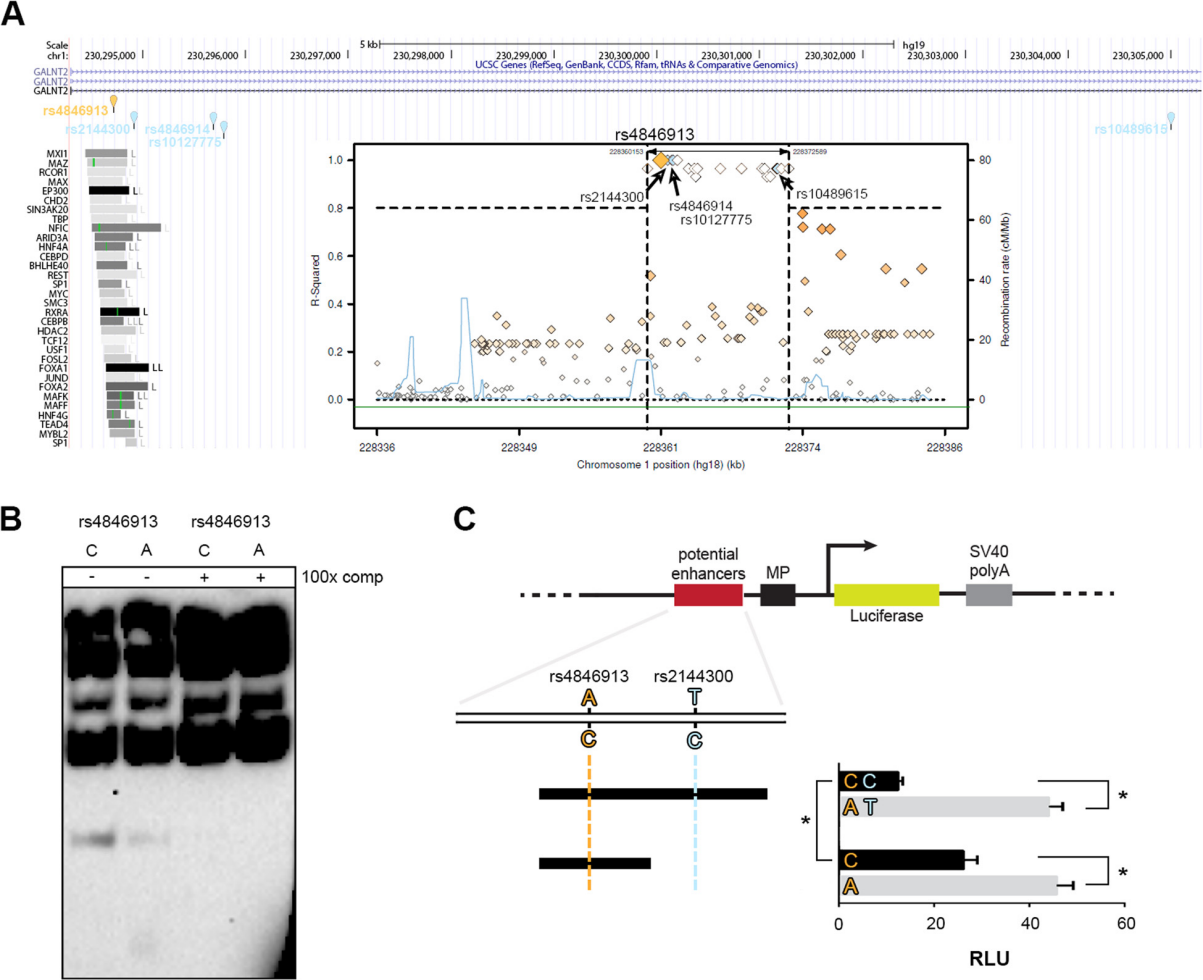
Functional effect of rs4846913 and rs2144300 at *GALNT2* in HepG2 cells. **a** UCSC Genome browser view of rs4846913 (orange) and associated GWAS-SNPs (cyan). The transcription factor ChIP-seq track from ENCODE shows the TF binding sites overlapping the different SNPs. The embedded regional LD plot (SNAP tool [11]) represents the genetic region around rs4849613 with SNPs in high LD (r^2^ > 0.8). The GWAS-SNPs associated are indicated in cyan. **b** EMSA for the C- and A-alleles of rs4846913. Lanes 3 and 4 represent a competition assay where a 100-fold molar excess of unlabeled probes was added to biotinylated probes and HepG2 nuclear protein extract. **c** Dual luciferase assay testing the enhancer activity of two constructs with the C- and A-alleles of rs4846913 and the C- and T alleles of rs2144300. * *P* < 0,001

Here we selected an AS-SNP in high LD with different GWAS SNPs associated to four traits related to phospholipids/cholesterol levels (Table 1). These are liver specific traits whose molecular events might be explained by the liver derived cell line HepG2, namely HDL-C and TG plasma levels associated to rs2144300, HDL-C associated to rs10489615, metabolite levels associated to rs10127775 and HDL-C, TG associated to rs4846914. Three out of four GWAS SNPs are located outside of TFBS annotated by ChIP-seq experiments, while rs2144300 is located on the edge of the regulatory element harboring rs4846913 (Fig. 1a).

**Table 1:** GWAS and AS-SNPs in high LD at *GALNT2*

Chr_id	Chr_pos (hg19)	AS-SNP	RegulomeDB^†^	In LD^‡^ with GWAS SNP	Associated traits
1	230294715	rs4846913	4	rs2144300^a^	HDL-C, TG
1	230294715	rs4846913	4	rs4846914^b^	HDL-C, TG
1	230294715	rs4846913	4	rs10489615^c^	HDL-C
1	230294715	rs4846913	4	rs10127775^d^	Metabolite levels

^†^ Regulome DB score as an index of regulatory activity [14]

^‡^ LD with r^2^ ≥ 0,8

^a^[3, 15]

^b^[4, 5, 16, 17]

^c^[18]

^d^[19]

We investigated the regulatory effect of rs4846913, an AS-SNP identified from ChIP-seq experiments in HepG2 cells (see Methods). This variant is located in the first intron of *GALNT2* and is in high LD (r^2^ > 0.8) with these four GWAS SNPs (Fig. 1a). The ENCODE project has performed ChIP-seq of the TF CEBPB in HepG2 cells and alignment of the reads to rs4846913 showed a genome-wide significant difference between the reference (C) and alternative (A) alleles.

We tested both alleles in EMSA and confirmed the differential binding with a stronger protein binding for the C allele (Fig. 1b). We then investigated the functional activity of both alleles using a dual luciferase assays. We found that the tested fragments showed a strong enhancer activity and a significantly lower signal for the C than the A allele suggesting that the TF that preferentially bind the C allele has a repressive activity. The tested fragments contain rs4846913 and rs2144300 (Fig. 1c, top fragment). To investigate if both SNPs are functional, we cloned rs4846913 separately in a smaller fragment and tested in a luciferase assay. We found a significant difference between the two alleles but the difference was significantly smaller compared to when both SNPs were present (Fig. 1c, bottom fragment). The functional activity of the rs4846913-A allele was the same for the small and large fragment indicating that it is independent of the genomic context (Fig. 1c). This suggests that a repressor binds to rs4846913-C allele and another repressor to the rs2144300-C allele. Analysis of motifs suggests that the TF ZBTB3 binds preferentially to the rs4846913-C allele and that TFs PLAGL1 and NR3C2 may bind to the rs2144300-C allele and act as repressors.

The genomic distribution of the GWAS SNPs (Table 1) supports the recent observation that several GWAS-SNPs can be associated to the same AS-SNP (e.g. rs4846913). Thereby a single functional AS-SNP can be causative and therefore the genetic driver of the association. This can be the case for several GWAS SNPs associated to one or more traits (Table 1) as observed previously for AS-SNPs in different cell lines [13]. This indicates that the statistical association to a trait in a GWAS may reflect the activity of one or more functional SNPs that have not been investigated, e.g. because they are rare or not genotyped.

## Conclusions

We propose that the AS-SNP rs4846913 and rs2144300 drive the association to HDL-C plasma levels through an inhibitory regulation of *GALNT2* rather than the reported GWAS SNP rs4846914. The luciferase assays data show a decrease in the functional activity of the construct harboring the C allele of the AS-SNP rs4846913, activity that was further lowered by the presence of the C allele of rs2144300 suggesting a synergistic inhibitory role and leading to the hypothesis that the low activity alleles rs4846913-C and rs2144300-C are preferentially bound by transcriptional repressors resulting in lower *GALNT2* levels and higher HDL-C.

## Abbreviations

AS-SNPs: allele-specific SNPs
GWAS: genome wide association studies
HDL-C: High-density lipoprotein cholesterol
LDL-C: low-density lipoprotein cholesterol
TF: transcription factor
TFBS: transcription factor binding site
TG: triglycerides
VLDL: very low-density lipoprotein
